# Lived experiences of nursing students about ethical concerns regarding mobile learning in educational and clinical contexts

**Published:** 2019-04-29

**Authors:** Hamid Reza Koohestani, Nayereh Baghcheghi, Mahmood Karimy, Morteza Hemmat, Morteza Shamsizadeh

**Affiliations:** 1 *Assistant Professor, Department of Medical Education, School of Medicine, Saveh University of Medical Sciences, Saveh, Iran. *; 2 *Assistant Professor, School of Nursing, Saveh University of Medical Sciences, Saveh, Iran. *; 3 *Associate Professor, Social Determinants of Health Research Center, Saveh University of Medical Sciences, Saveh, Iran.*; 4 *Assistant Professor, Department of Health Information Management, School of Medicine, Saveh University of Medical Sciences, Saveh, Iran. *; 5 *Instructor, Chronic Diseases (Home Care) Research Center, Hamadan University of Medical Sciences, Hamadan, Iran. *

**Keywords:** Mobile learning, Smartphone learning, Ethical concerns, Nursing student, Education, Clinical setting

## Abstract

The field of education has experienced a profound change following the introduction of mobile technology over the last decades, and nursing education is not an exception. This study explored the experiences of nursing students about the ethical concerns regarding the use of mobile devices for learning purposes, that is, mobile learning, in educational and clinical contexts. A qualitative phenomenological study was carried out on nursing students (n = 19) in Saveh University of Medical Sciences of Iran between December 2017 and April 2018. Data were collected through semi-structured interviews with open-ended questions. Data analysis was done using Colaizzi’s 7-step method, revealing four themes and nine sub-themes including: 1) preserving professional dignity (in front of the patient, and the teacher, and preserving academic virtual identity); 2) securing informed consent and respecting personal (the patient’s and teachers) autonomy; 3) proper and efficient use (observing the regulations and codes, and making educational use); and 4) avoiding harm (responsible use of class and patient data). It was revealed that using mobile technology in education could raise ethical concerns for nursing students, and this should be emphasized in nursing educational programs.

## Introduction

Mobile devises have emerged as new learning tools with a notable potential both in classrooms and outdoor learning environments (1). Mobile learning (m-learning) refers to using mobile electronic devices for educational purposes (2). In short, it means using a mobile handheld electronic device for learning without time or place limitations (3, 4). Portable devices are rightfully considered as powerful and interconnected handheld computers that among other things are used as efficient tools for learning and delivering targeted and curated content (5). M-learning has gained a more important role in improving the outcomes of learning and education (6). Mobile technology as a learning tool can add a new level of experience and significantly improve electronic learning (e-learning) appeal (7). 

Mobile devices such as smartphones can be integrated into health profession education (8, 9). Studies at the international level indicate that in general, university students have a positive attitude about using mobile devices for educational purposes  (10, 11). The findings of a systematic review showed that using an m-learning strategy in medical education can have positive effects on learning in all three domains of Bloom’s Taxonomy, that is the cognitive, effective, and psychomotor domains (12). There is a growing demand in medical science education programs for m-learning, since it is more convenient and readily accessible than traditional lectures (13). Gallegos and Nakashima (2018) reported that allowing the students to use mobile devises benefited the learning experience, and proper use of technology for educational purposes could enhance student interaction and engagement (14). 

Every day a new technological innovation is introduced in the field of higher education. The advancements have also affected nursing education. The integration of mobile technology has revolutionized learning over the past decades and it is widely incorporated into nursing education (15). 

M-learning has not only brought about deep changes in education, but it has also created new issues in the field of ethics. With the expansion of various aspects of nursing education, the dimensions of ethical concerns have also expanded. Use of mobile technologies by students in the educational context is not free of ethical concerns. While some studies have concentrated on ethical issued in m-learning, there is a paucity of studies to recommend ethical ways of using m-learning (16). The diversity of the usages of m-learning in nursing education also increases complexity of the issues.

Although several studies have been conducted to determine the effectiveness of m-learning in students’ academic achievements, there are no published study on the lived experiences of students about the ethical concerns surrounding the issue. There are universal ethical principles and values such as trustfulness, veracity, intrinsic dignity, respecting others’ privacy, freedom, honesty and the like; however, these concepts have different definitions in each situation. For instance trustfulness in business is different from trustfulness in education. 

All research projects start with a clear question that dictates the path and stages of the study. Thus, the research method is a function of the question. In this work, the question was “How do nurses experience the ethical concerns related to using m-learning in educational and clinical contexts?” As the question indicates, methodology should represent the nature of the phenomenon in a natural context along with the structures and factors that affect its development. Indeed, the authors expected to find the reality in its natural form as experienced by the students. In other words, the authors tried to perceive the experience instead of measuring it. Thereby, a qualitative method was the best option to illustrate the nature of the phenomenon in its natural context. There has been a paucity of qualitative studies on the lived experiences of nursing students about the ethical concerns regarding m-learning in educational and clinical contexts. Thus, the present study was an attempt to examine nursing students’ lived experiences about the ethical concerns related to the use of mobile devices for learning purposes in such contexts.

## Methods

A qualitative study was carried out using the phenomenological method of inquiry in Saveh University of Medical Sciences in Iran between December 2017 and April 2018.

Purposive sampling was used to identify and choose well-informed individuals in order to select the participants. Study inclusion criteria consisted of being an undergraduate nursing student, having passed at least two academic terms, and willingness to participate in the study. Given that nursing students entered the clinical setting starting the 2nd semester, all participants had at least 20 days of clinical experience. Nursing students with different demographic characteristics and clinical experiences (maximum variation) were included in the research for enhancing transferability of the results. However, to make sure that the participants were familiar with mobile technology, the research population was limited to undergraduate nursing students. These students were mostly at the same age group and could be recognized as the “digital native” generation. On the other hand, adding graduate nursing students could create unique landscapes.

Sampling was continued until data saturation was achieved and no new code was found. Data gathering was done using semi-structured interviews with open-ended questions (e.g. “Can you tell me about a situation where you used mobile phone for learning purposes?”, or “Tell me about your ethical concerns in m-learning experience”. The interviews were performed at the university campus (classroom, office, and any place of convenience). Given that the author is a research instrument in qualitative studies, guided questions were also obtained from the interview texts and used in subsequent interviews. Probing questions were used based on the information offered by the participations to gain deeper insight into the issue. To improve trustworthiness and rigor of the data, Guba (1981) was followed including the four criteria for credibility, transferability, conformability, and dependability (17).

To ensure creditability, the authors tried to create an intimate interaction with the subjects, win their trust by creating a relaxed and unstressed environment, and assign adequate time for data collection. In this way, the authors made sure that the participants could recount their experiences without tension. In addition, field notes, memos, member-check, and reviewing codes and themes by external reviewers (extended check) were used to guarantee the credibility of the data. To ensure conformability of the study, details of the study procedure including data collection, analysis, and extraction of codes and themes were elaborated so that the readers could form a judgment by reading the report. To support dependability, the whole procedure was extensively described to make it easy for readers to audit the study. With regard to transferability, a detailed account of the location of the research, interactions, and the processes observed during the study was also provided to facilitate judging the transferability of the study. The results were analyzed using Colaizzi’s 7-step method (18).

At first, the statements in the interviews were transcribed verbatim, and the transcriptions were read several times to obtain a common perspective with the participants. At the second stage, the key meanings and concepts were extracted and critical points were determined. At the third stage, the important themes were formulated and the authors tried to find the meaning of each extraction and the related concepts. The themes were clustered at the fourth stage, and the concepts were studied in detail and classified based on their similarity to the subject categories or the main concepts. As the fifth step, the results were used to achieve a comprehensive explanation of the subject. At this point, different subject categories of the same meaning were placed in larger categories to find the concepts of the main description. Afterward and as the sixth stage, the explanation of the intrinsic structure of the phenomenon under study was presented as an explicit statement of its basic structure. As the final step, creditability of the findings was ensured by carrying out a private interview with each participant. In the interview, each participant was asked to comment on the findings, and the results were then finalized. In observance of ethical codes, the subjects were informed at the start about research objectives, interview method, and confidentiality of their information, and were also assured that they could leave the study whenever they wanted. In addition, they were asked to sign an informed letter of consent. To ensure convenience of participation, the interviews were arranged in a way so as not to interfere with the daily routines and educational programs of participants. The author secured a permit from the Ethics Committee of Saveh University of Medical Science as well (IR.SAVEHUMS.REC1396.33). The students expressed their consent to participate in the study both orally and in written form. They were informed about the study purposes, interview method, confidentiality of information, and their right to participate in or leave the research at any point.

## Results

Among the 19 subjects in this study, 63.15% (n = 12) were female, and the average age of the participants was 20.57 ± 0.53. Moreover, three students were in the 3^rd^, five in the 6^th^, and six in the 8^th^ semester. 

Data analyses yielded 352 primary codes, which were then truncated based on overlaps and merged into 167 primary codes. After comparing the codes and their similarities, nine sub-themes and four themes were extracted (Table 1), which are presented below.

**Table 1:** The Main themes and sub-themes

Themes	Sub-Themes
**Preserving professional dignity**	Preserving professional dignity in front of the patient Preserving professional dignity in front of the teacher Academic virtual identity
**Securing informed consent and ** **respecting personal autonomy**	Securing informed consent and respecting the patient’s autonomy Securing informed consent and respecting the teacher’s autonomy
**Proper and efficient use**	Observing the regulations and codes Making educational use
**Avoiding harm**	Responsible use of class data Responsible use of patient data

***1.Preserving professional dignity***

*Preserving professional dignity in front of the patient*

The findings showed that it was very important for the students to preserve their professional prestige before patients. For this reason, while interviewing the patient or providing care, they would try to keep eye contact and refrain from using their mobile phones in any way that might be construed as disrespectful. In addition, some of the respondents said that using phones during care provision might interrupt the nurse-patient relationship and negatively affect the quality of rapport. One participant said: 

[“*Using any technology entails a specific code of conduct; for instance, I would never use my mobile phone while interviewing a patient, not even for educational or academic purposes. If I do so, it might be interpreted as disrespectful, and would be degrading to my professional prestige.”*] (Participant No. 11) 

Another one said:

[“*I would rather not use my phone while I interview the patients or provide care to them, because I think it is wrong and might lower my professional prestige. There are times when I try to take notes while interviewing or taking case history. In such situations I would ask for the patient’s permission beforehand to avoid any misunderstanding.”*] (Participant No. 8) 

*Preserving professional dignity in front of the teacher*

In this study, the participants’ concern about preserving their dignity before their teacher was also examined. Observing the codes of good conduct and decorum based on humanistic and professional values throughout academic and non-academic activities is a generally accepted standard among academicians. Using mobile phones in the presence of a teacher in clinical and educational environments, especially in a respectful manner, was one of the experiences mentioned by the participants. In observance of this rule, the students would limit the use of mobile phone for educational purposes to specific places and situations. One participant stated: 

[“*There is no doubt that mobile phones offer many possibilities regardless of time and place, but I do not use them everywhere. For instance, I would never check my phone while I am talking to my teacher, because it is unethical.”*] (Participant No. 12)

Another student said:

[“*Not all teachers are familiar with the advantages of smartphones for educational purposes. Many of them are aged and follow traditional methods so they reject the idea of using mobile phones for educational purposes. Respecting the teacher’s way is important for me and I would rather not use my phone before these teachers even for educational purposes.*] (Participant No. 2) 

*Academic virtual identity*

Accepting a role brings responsibilities and fulfilling them entails observing specific ethical codes. Being a student also requires observing specific ethical principles and codes. Among the students’ ethical concerns in the field of information technology was adopting a virtual identity. One participant said: 

[*“As a student, I try to have a proper online presence because I think the society has specific expectations from a student. For instance, I do not join just any channel or group in social media nor do I post careless messages or comments. Before forwarding a message or post, I make sure that it fits my professional prestige.”*] (Participant No. 4) 

Another student stated:

[*“Like all other forms of socialization, online socialization has its own codes and ethics. Although there is no direct physical contact in the virtual world, people still need to follow certain moral codes. I choose the words and stickers I use in social media very carefully because I believe prestige in the online world is even more important than the real world.”*] (Participant No. 19) 

***2. Securing informed consent and respecting personal autonomy***

*Securing informed consent and respecting the patient’s autonomy*

Informed consent is one of the main concepts in the field of medical ethics and patients’ rights worldwide. Similarly, patient autonomy is a basic element of healthcare, and respecting it is an accepted ethical principle. Respecting patients’ autonomy means acknowledging their right to accept or reject care. Entering patients’ private zone without permission is an unethical action. Smartphone features like recording film or voice make it much easier to neglect this ethical code, and failure to deal with this issue may lead to serious ethical challenges. One student said: 

[“*I always ask for patients’ permission before taking a picture of them, their file, or medical report. For instance, once there was a patient with diabetic foot. I asked him if I could take a picture of his foot and he accepted.”*] (Participant No. 17)

Another student said:

[*“Once I asked a patient for permission to film a grade 3 burn on his hand and the debridement process on the scar tissue for class conference, but he did not allow it. I totally understand why he did not; he was in a bad situation and had the right to refuse my request.”*] (Participant No. 9) 

*Securing informed consent and respecting the teachers’ autonomy*

Recording the teacher’s lecture for later use is very advantageous, since the student can listen or watch the lecture several times at a more convenient place. The practice, however, is not free of risk. Most of the participants said that voice recording in the class without permission is unethical; still, they had witnessed their classmates do so. One noted: 

[*“I think teachers have full autonomy whether or not to allow students to record their voice or image. Once one of the teachers was really upset that a student had recorded his voice and image in the classroom even though he had banned it.”*] (Participant No. 14)

***3. Proper and efficient use***

The participants’ experiences indicated that making good and fruitful use of mobile technology for learning purposes in the educational context was one of the ethical principles of m-learning. This theme is comprised of two sub-themes including observing the regulations and codes, and making educational use. 

*Observing the regulations and codes*

Educational regulations are deemed as organizational guidelines within the educational context that guarantee discipline order and discipline. Failure to observe the laws or breaching them for personal benefits shows lack of respect for educational environments and neglect of ethical organizational principles. One student noted: 

[*“I always try to respect the laws concerning mobile use in educational environments. Like the real world, the virtual world should be used based on specific rules and regulations. Failure to respect these rules and regulations results in harm to oneself and others. For instance, according to the regulations, we must put our mobile phones on silent mode in the classroom, and that is what I always do.”*] (Participant No. 13) 

Another participant said:

[*“The laws in this respect are very important for me; for instance, if a teacher does not let the students use their mobile phones in the classroom or during a training course, I will not disobey. I will not use my mobile phone if it is not allowed, since it is illegal and unethical to do so.”*] (Participant No. 6) 

*Making educational use*

The main feature of mobile learning that is generally emphasized in the literature is the learning possibility regardless of place and time. Using m-learning, students can access educational material of any kind in classrooms and clinical contexts. Making fruitful and educational use of mobile phones in class or while providing care to patients was one of the ethical considerations mentioned by the participants. One student said: 

[*“For example, if the teachers allow using mobile phones to search for academic material in the class, I will use my phone only for that purpose and on that occasion. I think it is unethical to make other uses of your phone in the classroom.”*] (Participant No. 1)

Another student said:

[*“If I use my mobile phone while interviewing a patient, it will be only for educational purposes.”*] (Participant No. 3)

***4. Avoiding harm***

*Responsible use of class data*

Recording lectures and class audio creates a valuable educational resource for students that provides better understanding on a personal level, and can also be shared with other students and audiences. Observing ethical codes in terms of using and sharing videos and voices of teachers or other students, however, was another concern for the participants. 

The majority of the participants believed that sharing content is ethical only when recipients of a recording personally attended an event. Therefore, it is unethical to share it with others and it might harm those who participated in the event. 

The students’ experiences indicated that they were faced with certain ethical challenges in this field. One participant said: 

[*“A friend of mine once shared part of a video on YouTube in which our teacher asked a question and another student gave a rather irrelevant and somewhat funny answer. This action created a quarrel, and I think it was quite unethical.”*] (Participant No. 11)

Another participant said:

[*“Following a disagreement with the teacher, one of my friends played an audio file that he had recorded at the beginning of the semester for the education department to prove that the teacher was not doing what he had promised before. I found it quite unethical and personally would never do it.”*] (Participant No. 18)

*Responsible use of patient data*

A key element of observing patient’s rights is to respect their privacy, and in terms of information, confidentiality of patient data is of particular importance. The Patients’ Rights Charter puts special emphasis on protecting patients’ information. Sharing patients’ information can be interpreted as violation of their rights, whether it is a lab report or a video file. As stated by one participant: 

[“*Films and pictures taken from patients or their medical files should be treated with care. I will never publish films or pictures that I have taken with the consent of the patient on the Internet. If I share a photo of a patient’s chest or electrocardiogram, I will make sure that the identity of the patient remains unknown.”*] (Participant No. 5) 

Another student said:

[*“Unfortunately and in some cases, irresponsible use of mobile phones has turned the private issues of patients into a public matter. My friend once filmed an interview and the medical history review process for educational purposes with the permission of a cardiac patient. Later he published that film publicly, which I believe was unethical.”*] (Participant No. 10)

## Discussion

While new technologies, including mobile technology, can act as a positive force to facilitate change, they can also be a source of new ethical challenges. The findings showed that using mobile technology in clinical and educational contexts causes ethical issues, and it is critical for nursing students all around the world to ponder on these issues in their daily education. 

The rapid growth of mobile device use over the recent years has led to conflicts with the accepted standards of behaviors (19). Still, the wide diversity of contexts where m-learning can be used adds to the complicacy of the issue for nursing students. The participants mentioned complicated and dynamic experiences while using their mobile devices. Learning in the hospital context also creates another layer of complexity for individuals about how and when they are ethically allowed to use their devices before patients and teachers.

Four themes were extracted from the students’ experiences with regard to the ethical concerns associated with m-learning: preserving professional dignity, securing informed consent and respecting personal autonomy, proper and efficient use, and avoiding harm. To the best of the authors’ knowledge, the present research was the first qualitative study on the lived experiences of nursing students about the ethical concerns related to m-learning. It is notable, however, that m-learning has been qualitatively and quantitatively examined in some studies as a general issue to determine the effective factors in accepting the technology or its learning effect on students. 

For instance, Xiao et al. (2017) reported that nursing students had a positive attitude and understanding about m-learning. They concluded that the use of m-learning by students should be promoted by providing suitable conditions (20). Iqball and Qureshi (2012) studied students’ perception of m-learning adaptation and reported that “practicality, convenience, and accommodating settings” were significant factors in the students’ desire to use m-learning (21). Yoo and Lee (2015) illustrated that mobile applications may render as highly effective simulators which may provide an exactly precise image of human patients for the purpose of teaching cardiopulmonary assessment skills (22). However, the ethical concerns related to m-learning in nursing education have been neglected by researchers.

Developing one’s knowledge is an essential part of learning, and in clinical settings, students tend to use mobile devices for rapid acquisition of information. Still, some of the participants said that they did not use their phones before patients, as they perceived it a rude action. This concern was also mentioned by patients and hospital personnel (23).

Respecting patients’ autonomy and gaining their informed consent was another theme extracted from the students’ experiences. Capturing digital content about patients without prior consent (for instance picturing embarrassing situations and neglecting their intellectual property) and sharing it on the Internet may create serious ethical challenges, even if done unintentionally. The purpose of securing permission beforehand is to make sure that the patient or surrogate is given the chance to accept or reject the action or treatment under no duress. Otherwise, autonomy and dignity of the subjects is neglected (24).

Therefore and as mentioned by the majority of the students, recording information and content about patients should be done only after obtaining their free and informed consent. Irresponsible use of patients’ electronic information and digital educational content was another ethical concern extracted from the experiences of the participants. 

One ethical concern with regard to the risk of information accessible only for a limited audience was public distribution of that information. Although most of the participants agreed that publishing such information without permission was unethical, some had experienced ethical challenges in this regard. Other studies have demonstrated that irresponsible and unethical use of information and educational materials may be an ethical concern with regard to m-learning (25). 

The findings indicated that one consequence of using patients’ information irresponsibly was rooted in capturing and disseminating videos that might go viral. Some students film their teachers or classmates and publish the videos on the web, which in most of the cases is done to humiliate them. Therefore, along with providing a supportive environment for mobile phone use in class, teachers should play as ethical role models to prevent cyber-bullying. 

Mobile learning is widely practiced in universities as a sort of educational method (2, 8); however, the results of this study showed that some students do not respect the professional ethics associated with the use of mobile technology and find the cyber world a place to act beyond ethical limits. 

One probable problem is the lag between the rapidly advancing technology and the slow evolution process of the rules on its use. Mobile devices have become a part of students’ daily lives, nursing students included. Teachers are required to adapt to the technology and modify their teaching techniques to facilitate ethical m-learning. Likewise, students should be equipped with the knowledge that using mobile devices may have negative effects on their professional prestige before teachers and patients. Ethics is an ambiguous concept (24) and the results of this study indicated that many students have a poor understanding of the ethical way to use mobile devices in teaching and learning contexts. As a result, one may conveniently slip into a sort of ethical relativism and choose to follow one’s own path (19). Lack of training, which is the case in many situations, makes it hard for the students to find an ethical way to manage their use of mobile devices for educational purposes. 

In our path toward a more ethical approach to m-learning, our focus must be shifted from mobile devices to the issues that are the real concerns. The main question is how to use mobile devices in learning. Therefore, rather the prohibiting their use, we need approaches to limit bad behaviors and empower the students with efficient ways to deal with inappropriate behaviors of others. 

Like other fields of education, improvement of student-teacher-patient relationship in terms of observing the ethics of m-learning leads to an enhanced quality of nursing education. By relying on the principles of ethics and extending their knowledge in this regard, students can use m-learning in a more intelligent, purposeful and informed manner. University teachers also need to pay special attention to ethical codes on using mobile devices both in the real and online world.

As a qualitative research, the findings cannot be generalized to a larger population, but may be transferable to similar environments. However, our research was the first of its kind to explore nursing students’ experiences regarding the ethical concerns related to using mobile devices for learning purposes in educational and clinical contexts. Future studies with different students, schools and countries are needed to investigate the role of additional constructs in the issue.

## Conclusion

Using mobile technology in clinical and educational contexts can raise ethical concerns for nursing students. Rather than prohibiting the use of mobile devices, we must devise approaches to restrict unacceptable behaviors and help the students develop strategies to deal with others’ inappropriate behaviors. This should be emphasized in nursing educational programs. Additionally, it is necessary to adjust teaching and education and allow the students to use their mobile devices in a more appropriate manner.

## Notes:

**Citation to this article:**

Koohestani HR, Baghcheghi N, Karimy M, Hemmat M, Shamsizadeh M. Lived experiences of nursing students about ethical concerns regarding mobile learning in educational and clinical contexts. J Med Ethics Hist. 2019; 12: 5.
